# Membrane Recycling: Exploring Ozone as a Viable Alternative to Chlorine for Polymeric Membrane Transformation

**DOI:** 10.1021/acsestengg.5c00517

**Published:** 2025-07-31

**Authors:** Bianca Zappulla-Sabio, Lide Jaurrieta, Wolfgang Gernjak, Harikrishnan Balakrishnan, Ludovic F. Dumée, Hèctor Monclús, Gaetan Blandin

**Affiliations:** † LEQUiA, Institute of the Environment, 16738 University of Girona, Girona 17003, Spain; ‡ 504638 Catalan Institute for Water Research (ICRA), Emili Grahit 101, Girona 17003, Spain; § Catalan Institution for Research and Advanced Studies (ICREA), Passeig Lluís Companys 23, Barcelona 08010, Spain; ∥ 105955 Khalifa University, Research & Innovation Center for Graphene and 2D Materials (RIC-2D), Abu Dhabi 127788, United Arab Emirates; ⊥ Khalifa University, Department of Chemical and Petroleum Engineering, Abu Dhabi 127788, United Arab Emirates; # Element Zero, Research and Innovation Department, Malaga, Western Australia 6090, Australia; ∇ Nanjing Tech University, Nanjing 210009, China

**Keywords:** chlorine, membrane degradation, membrane recycling, ozone, polymeric membranes

## Abstract

Ozone, a strong oxidant, induces oxidative degradation in various materials and is known as an effective chemical for polymer modification. This study assesses ozone as an alternative to chlorine oxidation for converting end-of-life reverse osmosis membranes into nanofiltration- and ultrafiltration-like membranes across various new and used reverse osmosis and nanofiltration membranes. Membranes were characterized in terms of permeability and salt rejection, as well as surface characterization. Experiments were conducted at high ozone exposure (20 ppm) and low ozone exposure (3 ppm). At high exposure, ozone was found to degrade both the polyamide (PA) and polysulfone (PSf) layers, opening new possibilities for polyester (backing layer) recycling. At low exposure, degradation was limited to the PA layer; ozone converted membranes more effectively than chlorine, achieving similar performance in less time and at lower doses—75 and 225 L·m^–2^·h^–1^·bar^–1^ for SW and BW membranes after 30 min at 3 ppm ozone, comparable to 6000 ppm chlorine over 50 h. Ozone significantly impacted NF90, raising the permeability to 150 L·m^–2^·h^–1^·bar^–1^ in 15 min at 3 ppm, while NF270 remained more resistant at 35 L·m^–2^·h^–1^·bar^–1^. Ozone caused patchy degradation due to bubble interactions, while chlorine led to uniform attack. These findings highlight ozone’s potential as a viable and more sustainable alternative to chlorine for polymeric membrane transformation.

## Graphical abstract

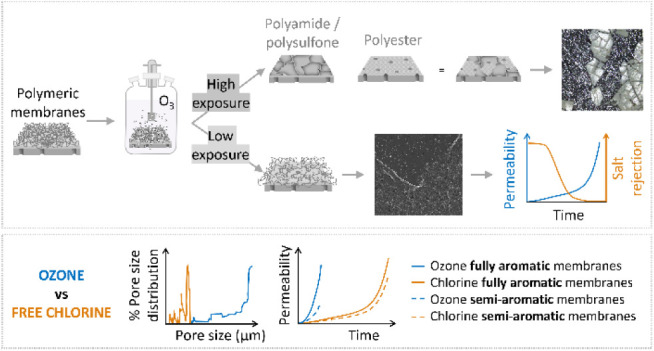

## 1 Introduction

The growing imbalance between clean water demand and supply has led to economic water shortages affecting a quarter of the global population.^1^ This challenge underscores the urgent need to develop sustainable water production technologies. In recent years, desalination has gained significant interest as an effective solution, capable of producing clean water from seawater and brackish sources.^1,2^ However, reverse osmosis (RO) membranes, a key technology in desalination, have a limited operational lifespan of less than 10 years.^1,2^ In 2022, this resulted in the disposal of around 35,000 tons of end-of-life (EoL) polymeric membranes, most of which were either incinerated or sent to landfills.^2^ Given the large volume of discarded membranes, finding alternative strategies for their reuse has become increasingly important. Studies have shown that EoL RO membranes can be repurposed into less restrictive filtration membranes by selectively degrading their polyamide (PA) layer.^3,4^ Thin-film composite (TFC) membranes have a three-layer structure: a selective layer known as PA, a support layer that is normally a polysulfone (PSf) one and a backing layer of polyester (PET).^5^ Chemical treatments using solvents such as sodium hypochlorite, potassium permanganate, and hydrogen peroxide have proven effective in partially or completely removing the PA layer, enabling the transformation of RO membranes into nanofiltration-like (NF-like) or ultrafiltration-like (UF-like).^6–10^ This approach offers a promising pathway toward extending membrane life cycles and promoting sustainable membrane management.

Sodium hypochlorite is employed in membrane processes as an effective method to convert TFC RO membranes into NF-like and UF-like membranes. Under high chlorine exposure, the membrane’s performance properties can be altered, enabling the development of membranes for new applications.^11–18^ NF-like membranes are produced through partial degradation of the PA layer, while UF-like membranes require complete degradation.^3,4^ This transformation occurs due to the interactions of the hypochlorite with the functional groups of the PA layer, such as amide groups (−CONH−) and aromatic rings.^6^ Other methods, such as potassium permanganate, hydrogen peroxide, or UV treatment, have been investigated in previous studies as potential alternatives to chlorine for converting RO membranes.^8–10^ However, these methods have not gained significant traction due to their inherent limitations (e.g., accessibility to the membrane inside the module, chemical consumption, etc.) when compared to the widely adopted free chlorine method. Ozone may not present these limitations, as it could be in contact with the membrane without requiring module disassembly. For instance, ozone can be predissolved in water and circulated through the module at high crossflow velocity or introduced in the form of ozone-enriched air bubbles passed through the module.

Ozone is an allotrope of oxygen composed of three oxygen atoms in a gaseous state, known for its powerful ability to directly oxidize organic compounds and as a strong oxidant capable to reduce the amount of foulant species in wastewater treatment.^19,20^ This factor, along with the need to minimize membrane fouling, has positioned ozone-based advanced oxidation processes as some of the most competitive technologies for drinking water and wastewater treatment. Ozone can be applied upstream of RO membranes to mitigate fouling and enhance overall system performance.^21,22^ Additionally, ozone induces oxidative degradation in various materials and is widely recognized as an effective method for modifying polymer surfaces, making it a valuable tool for membrane treatment and performance optimization.^23–25^ However, questions remain regarding the impact of residual ozone on the polymeric structure of downstream RO membranes and the potential for controlled ozone application to facilitate the targeted membrane surface transformation.

A recent study evaluated the resistance of various NF polymeric membranes to exposure to dissolved ozone, focusing on the heightened sensitivity of polymers containing C=C double bonds.^19,20^ The findings revealed that ozone caused significant degradation of the PA layer, whereas the PET layer showed no notable degradation. This difference can be partially attributed to the PET layer’s strong resistance, as its aromatic rings are shielded from electrophilic attack by the presence of strongly electron-withdrawing sulfone groups. Overall, ozone degradation of RO membranes remains poorly studied and, as it has been done for chlorine, more work is required to (1) avoid premature degradation during membrane operation if ozonation is implemented as pretreatment and (2) reversely define if controlled ozonation of RO membranes can be used for EOL membrane transformation.

Given the existing gap in knowledge regarding the impact of ozone on polymeric membranes, this explorative study aims to evaluate its impact on the layers of thin-film composite materials and its potential usage as an alternative method to hypochlorite to convert polymeric membranes. A protocol was developed to evaluate the effectiveness of ozone on both RO—new and used—and NF commercial membranes regarding different contact times and compared to hypochlorite treatment. Permeability and salt rejection tests were carried out with complementary surface characterization, such as field-emission scanning electron microscopy (FE-SEM) and Fourier transform infrared (FTIR). All these experiments allowed us to gain a deeper understanding of the changes happening on the membrane after being exposed to ozone and to develop a new systematic method to convert EOL membranes for successful reuse, thus moving toward a circular economy.

## 2 Materials and Methodology

### 2.1 Materials

#### 2.1.1 Membranes

New commercial membranes—SW30HRLE, BW30, NF90, and NF270—referred to here as SW30, BW30, NF90, and NF270, respectively, were purchased from DWS Octochem, DuPont (Vandalia, Illinois, USA). The membranes were cut into coupons with a surface area of 0.014 m^2^ using a standardized cutting pattern. Similarly, used SW30HRLE-400-ilec and BW30–400-FR membrane modules, sourced from industrial applications and referred to here as SW30-used and BW30-used, were opened in the laboratory, and membrane sheets were cut following the same pattern. All membranes, both new and used, were stored at 4 °C in distilled (DI) water.

#### 2.1.2 Chemicals

Sodium chloride (NaCl), sodium hydroxide (NaOH), sodium bicarbonate (NaHCO_3_) and sodium hypochlorite (NaClO) were purchased from Sigma-Aldrich (Barcelona, Spain). DI water was obtained from a Millipore purification system.

### 2.2 Ozone Membrane Exposure and Setup

The aim of this study was to evaluate the impact of ozonation on RO and NF membranes under severe and then milder conditions. The experimental design was based on a previous study focused on NF membranes, where a 10 ppm ozone concentration was applied for 1 h.^19^ The current study was conducted in two steps. At first, an evaluation of membrane layers’ resistance to intense ozonation under high dosage (20 ppm) and long contact time (up to 3 h) was performed. Small membrane coupons were used since only surface characterization was conducted on the treated membranes. This approach facilitated the rapid screening of a large number of samples, allowing for the assessment of physical changes after intense treatment and the identification of general damage patterns. Based on the significant degradation observed in the first set of tests, the second phase was designed with more moderate ozone dosages (3 ppm) to better control the chemical attack, targeting membrane conversion rather than degradation. Larger samples were exposed to ozone to allow for permeability and rejection tests in addition to surface characterization. This phase also included a comparison between RO and NF membranes and a comparison with sodium hypochlorite treatment.

#### 2.2.1 Ozone Setup

As shown in Figure 1, a 3 L reactor was filled with DI water and enriched with ozone produced by an ozone generator (ANSEROS Ozone Generators, model COM AD-04, Germany). To prevent liquid backflow into the ozone generator, a 1 L washing bottle was placed between the generator and the reactor as a protective measure. Downstream of the reactor, another 1 L washing bottle containing a 1 M NaOH solution was added to decompose ozone before it was vented into the fume hood. An ozone sensor was installed near the setup throughout the experiment to monitor for potential ozone leaks. Samples of the ozone-enriched water were collected using a pipet and analyzed using the indigo method with a spectrophotometer at 600 nm. In the low-exposure-time experiment, the pH from the solution was stabilized at 8 by adding 3 mM NaHCO_3_ at the beginning of the experiment.

**Figure 1:**
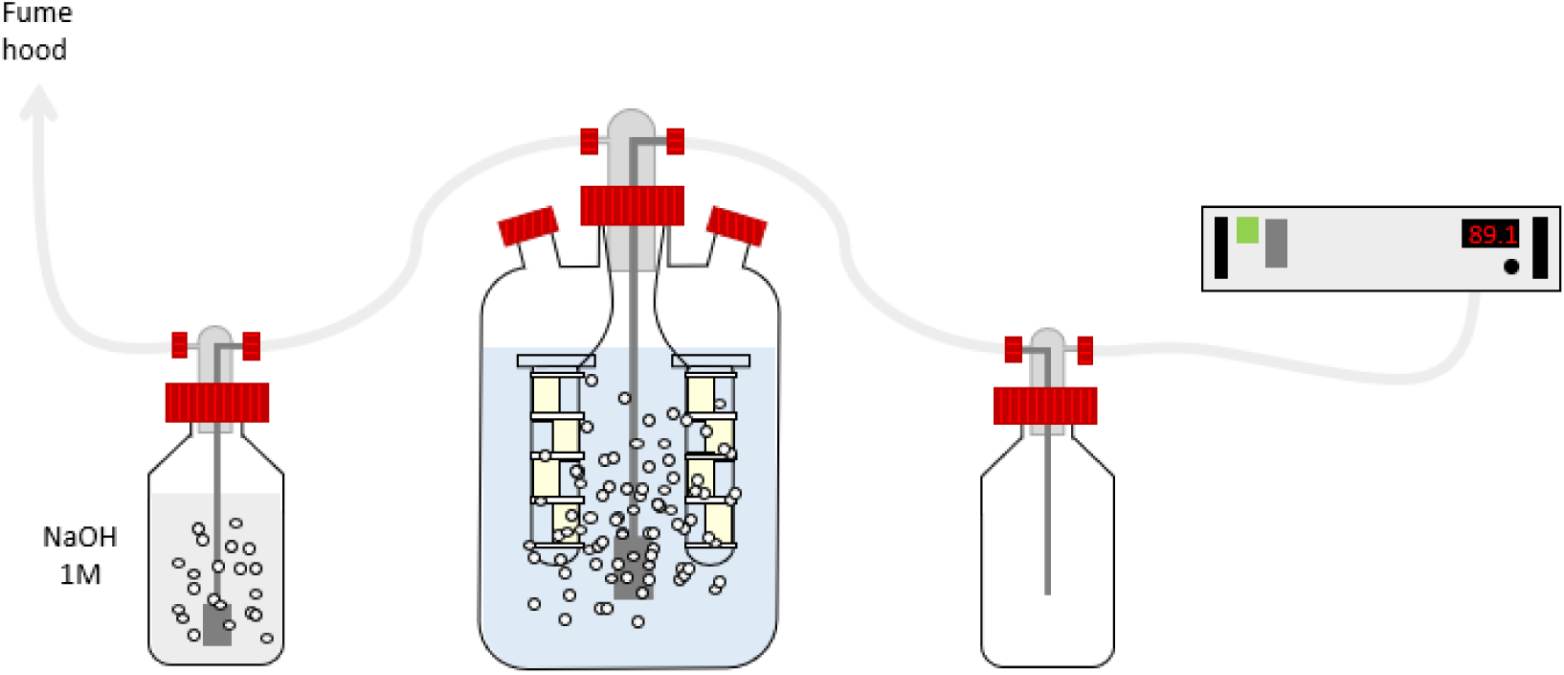
Ozone setup used in the high-exposure-time experiment. At low exposure time, the setup was the same, but membranes were placed inside the 3 L reactor without being wrapped around a glass tube and secured with plastic ties.

#### 2.2.2 Ozone High-Exposure Membrane Conversion

Small RO membrane coupons (2 cm × 2 cm) were immersed in a 20 ppm ozone-enriched water solution for 30 min, 1 h, and 3 h. Efforts were made to ensure that the active layer of the membranes was fully exposed to ozone. To achieve this, membrane coupons were wrapped around a glass tube and secured with plastic ties (Figure 1). The ozone concentration and pH of the solution were measured at the beginning and after each sample was removed to allow estimation of ozone exposure. After exposure, coupons were rinsed with DI water and stored until further characterization.

#### 2.2.3 Ozone Low-Exposure Membrane Conversion

Larger RO—new and used—and NF membrane coupons with a surface area of 0.014 m^2^ (to allow for permeability and rejection tests) were submerged in a 3 ppm ozone-enriched water solution for 1-, 5-, 15-, and 30-min exposure. The pH of the solution was maintained at 8 by adding 3 mM NaHCO_3_ at the start. Ozone concentration and pH were measured at the beginning of the experiment and after removing each sample. Following exposure, the coupons were rinsed with DI water and stored until further testing for permeability, salt rejection, and surface characterization.

### 2.3 Chlorine Membrane Exposure

As a baseline to assess the impact of chlorine on RO and NF membranes, the PA layer was removed through a three-step chemical oxidation protocol. First, the membranes were soaked in a 6000 ppm free chlorine solution for 1 h (exposure dose of 6000 ppm·h) and characterized. Then, an additional 4 h soaking to achieve the total dose of 30,000 ppm·h was applied to convert the RO membranes into NF-like membranes.^3,4^ The converted membranes were then characterized to evaluate their performance. Next, the coupons were soaked for an additional 45 h, achieving a total dose of 300,000 ppm·h, which is the dose known to convert the EOL RO membranes into UF-like membranes.^3,4^

### 2.4 Membrane Characterization

#### 2.4.1 High-Pressure Lab-Scale Filtration Setup

A high-pressure lab-scale filtration setup was used to characterize membranes under operating conditions of 15 bar with a 2 g·L^–1^ NaCl feed solution. The system utilized a 20 L feed solution tank (SETPAR Export, model Fontsere, Spain) maintained at 20 ± 0.5 °C. The solution was pumped at a flow rate of 2 L·min^–1^ using a high-pressure piston pump (CAT Pumps, model 3CP1231, USA) to a filtration cell (Sterlitech, model Sepa CFX Cell, USA) with an effective filtration area of 0.014 m^2^. Pressure was regulated via a back-pressure valve on the retentate line, and the permeate stream was recirculated into the feed tank. Permeate flux was determined by monitoring mass changes over time using a Kern PCB 6000-1 balance. Conductivity measurements of samples, collected every 10 min, were performed using a conductometer (Crison Instruments meter, model BASIC 30, Spain) with the samples subsequently returned to the feed tank. Additionally, weight and pressure data were continuously monitored using a Bluetooth-enabled Arduino-based datalogging system.

#### 2.4.2 Attenuated Total Reflectance Fourier Transform Infrared (ATR-FTIR)

ATR-FTIR spectroscopy analysis was performed using a Bruker Alpha FT-IR spectrometer instrument with a flat plate crystal as the attenuated total reflection (ATR) element. ATR-FTIR was equipped with a deuterated triglycine sulfate (DTGS) detector element to provide a higher sensitivity. Spectra were recorded in the range of 400–4000 cm^–1^ with a resolution of 4 cm^–1^. ATR-FTIR was employed to detect membrane layer degradation following membrane conversion and to assess the potential impact of ozone on the membrane composition. The analysis focused on key spectral bands characteristic of PA and PSf layers. The characteristic bands analyzed for the PA layer included N–H stretching (3300 cm^–1^), C=O stretching from the −CONH radical (1668 cm^–1^), and N–H bending (1584 cm^–1^). For the PSf layer, the key bands were C–H stretching (1417 cm^–1^), S=O stretching (1147 cm^–1^), and C–S stretching (555 cm^–1^).^26–29^

#### 2.4.3 Field-Emission Scanning Electron Microscope (FE-SEM)

The FE-SEM membrane analyses were performed using a field-free analytical UHR SEM, the TESCAN CLARA model. The samples were air-dried prior to being placed on the sample holder and later coated with a silver alloy to minimize charging effects and offer excellent charge dissipation. Subsequently, the samples were coated with a thin layer of carbon via sputter coating to ensure surface conductivity. Images were acquired at an accelerating voltage of 5 keV and a working distance of approximately 10 mm. Various magnifications were applied to analyze the membranes: 500× to observe the overall membrane structure before and after treatment, and 10 000× and 30,000× to assess the extent of PA layer degradation in greater detail. These magnifications were selected to provide a detailed examination of the membranes at various scales, offering insights into their overall structure and highlighting any changes resulting from ozone exposure.

#### 2.4.4 Laser Profilometry

Surface roughness and surface profile data were acquired through the utilization of the LEXT OLS4100 3D laser microscope, produced by Olympus, Australia. This equipment employs laser scanning to conduct noncontact 3D measurements of intricate surface features. Measurements have been carried out on small (5 × 5 μm) and large surface areas (50 × 50 μm). Small surface areas are focused on local surface roughness (polyamide structure), while large areas are focused on the heterogeneity of the whole sample.

#### 2.4.5 Liquid–Liquid Perm-Porometry

Pore feature analysis, involving the evaluation of the average pore size, pore size range, and distribution, was executed utilizing the INNOVA 500 liquid–liquid porometer, manufactured by Poretech Instruments Inc., Taiwan.

## 3 Results and Discussion

### 3.1 Initial Assessment: Ozone High-Exposure-Time Conversion

The protocol was initially tested using both new and used RO membranes (Figure 2a) to determine the optimal operating conditions. The effects of ozone exposure at varying durations were evaluated. Figure 2 presents the initial visual inspection of the membranes before (b) and after (c,d) ozone treatment. A noticeable color change—from yellowish to completely white—was observed on all membranes after the shortest exposure time (30 min at 20 ppm), qualitatively evidencing the effect of ozone on the membranes.

**Figure 2:**
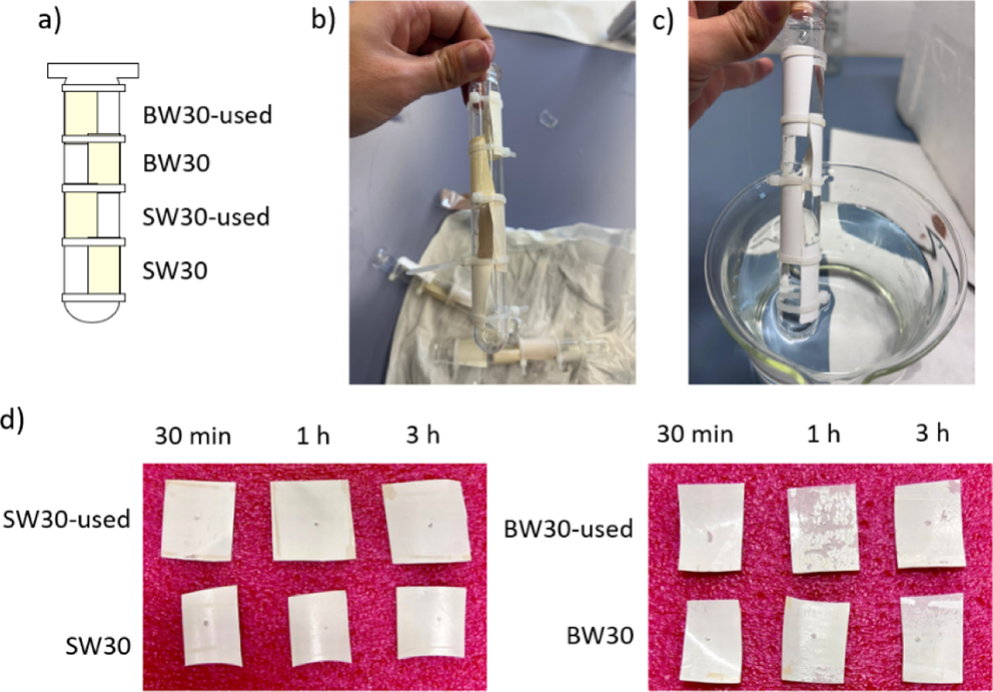
Membranes under high ozone exposure: (a) membrane distribution, (b) membranes before treatment, (c) membranes after 30 min of ozone treatment, and (d) membranes after different ozone exposure durations.

To investigate the compositional differences between the two RO membrane types and to compare the membranes before and after ozone treatment, SEM, FTIR, and profilometry analyses were performed on all samples.

SEM images (Figure 3) display the membranes before treatment and after 1 h of ozone exposure at 20 ppm. Extensive cracks were observed across the entire membrane surface, indicating a severely compromised structure. Notably, for the BW30-used membrane after 1 h of treatment (Figure 3 h), the PET layer became visible, confirming the complete removal of both PA and PSf layers. These findings are consistent with the visual inspection, which already revealed visible cracks across the surface and the complete removal of the selective and support layer. Differences between new and used BW membranes were observed, with a more pronounced impact on the used membranes. This may be attributed to membrane aging, which increases susceptibility to chemical interactions. Moreover, differences between BW and SW membranes can be attributed to the lower degree of PA cross-linking in BW membranes, which generates a higher concentration of −OH groups, rendering the membrane more susceptible to ozone attack confirming a compositional difference between the two RO membrane types.^30,31^

**Figure 3:**
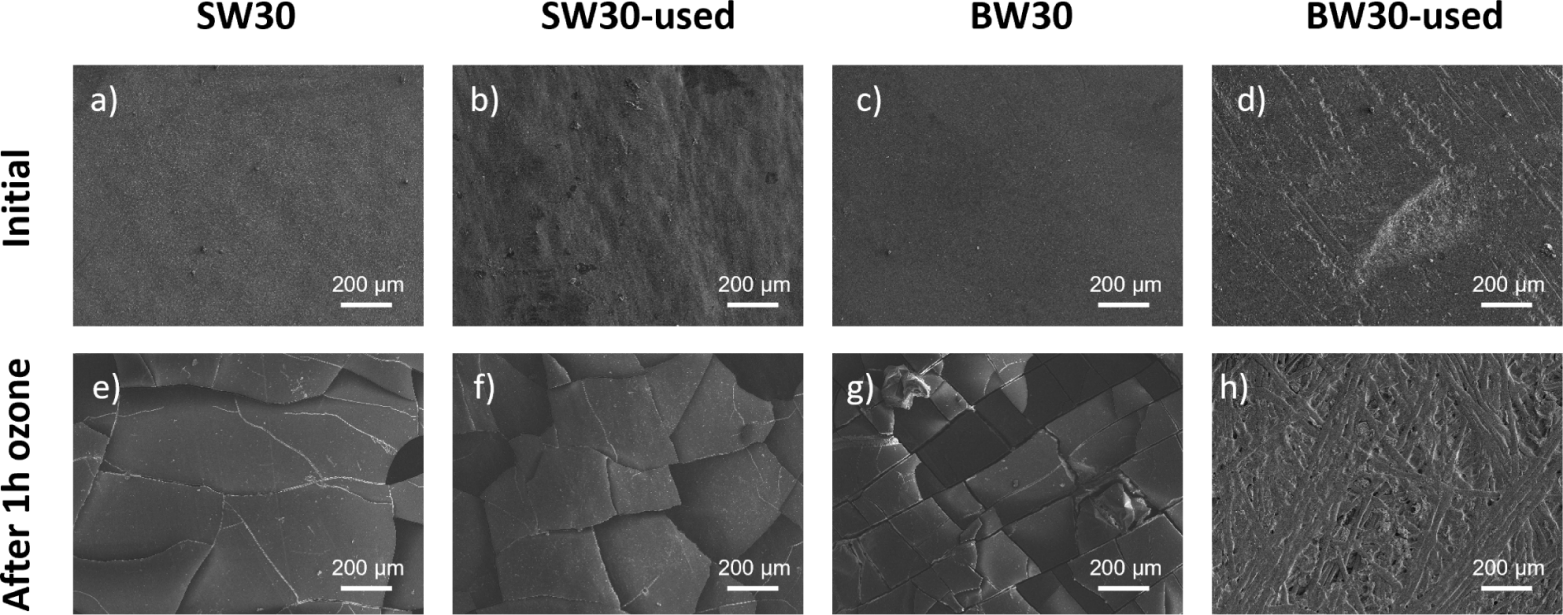
FE-SEM images before and after 1-h ozone treatment: (a,e) SW30, (b,f) SW30-used, (c,g) BW30, and (d,h) BW30-used.

Profilometry tests were conducted for each membrane, and the results are presented in Figure 4. After 30 min of treatment, the membranes did not exhibit significant surface damage, although the initial formation of cracks became noticeable. Following 1 h of exposure, the BW30-used membrane showed a substantial surface alteration with partial removal of the PA layer, revealing the underlying structure. After 3 h of treatment, SW membranes displayed extensive cracking across the entire surface, while BW membranes exhibited severe degradation with the PET support layer fully exposed. These findings are consistent with the damage observed in the SEM analysis.

**Figure 4:**
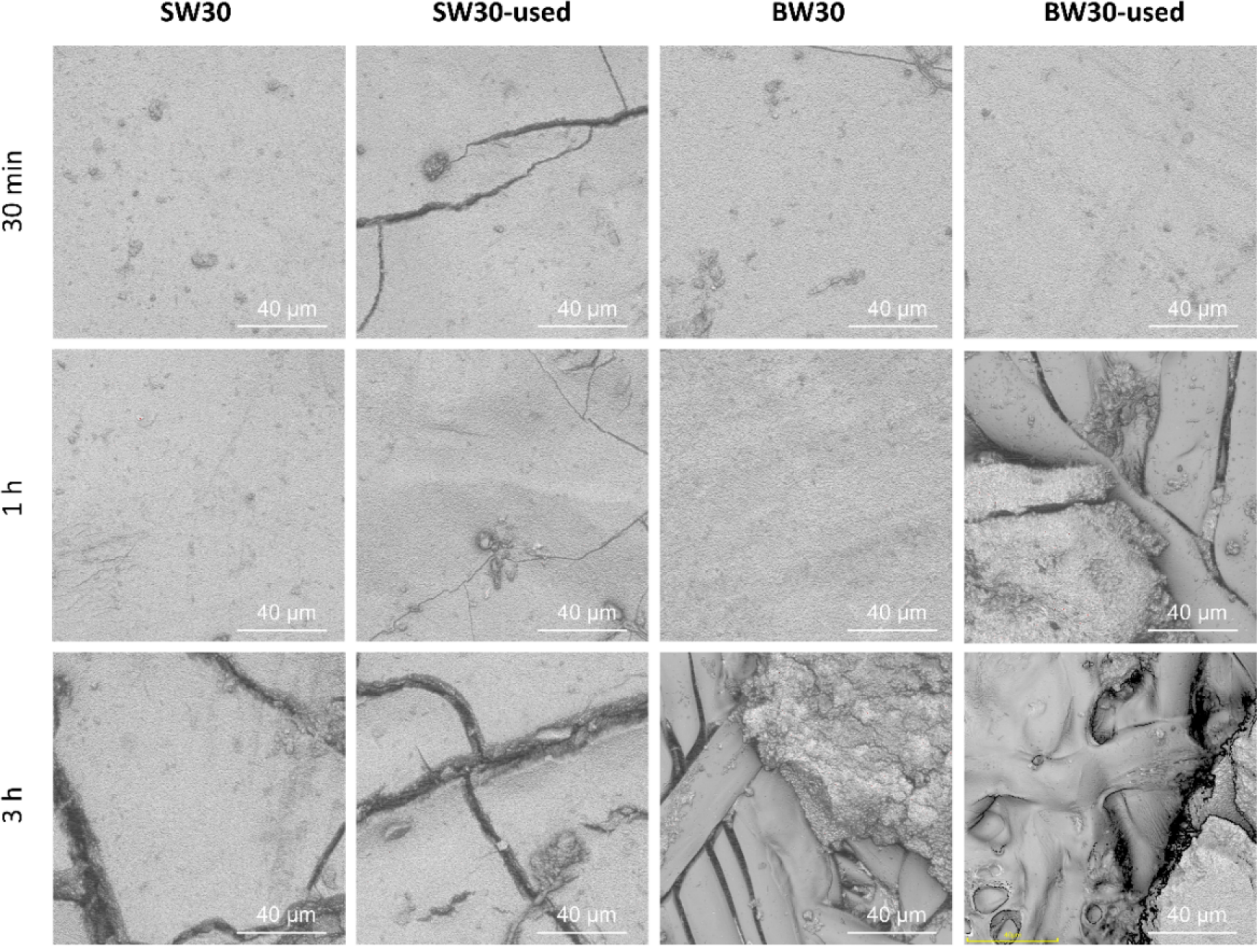
Profilometry test for the high-exposure membranes after 30 min, 1 h, and 3 h treatment.

The most relevant FTIR bands for the PA layer (N–H stretching—3300 cm^–1^, C=O stretching from the −CONH radical—1668 cm^–1^, and N–H bending—1584 cm^–1^) and for the PSf layer (C–H stretching—1417 cm^–1^, S=O stretching—1147 cm^–1^, and C–S stretching—555 cm^–1^) are presented in Figure 5 as the percentage of change between the untreated membranes and those treated for 30 min, 1 h, and 3 h. The full spectrum is presented in Figure S1. As expected, the PA bands were the most significantly impacted by ozone, with reductions ranging from 20% to 95%, with the N–H bending band being the most affected. Interestingly, the PSf bands also showed degradation, with significant reductions in the S=O (1147 cm^–1^) and C–S (555 cm^–1^) bands, indicating that ozone impacts not only the PA layer, as initially expected, but also the PSf layer. C–S bands were reduced by nearly 60%, and S=O bands were reduced by a maximum of 30%. These results demonstrate the progressive removal of both the PA and PSf layers. This conclusion is supported by the limited penetration depth of infrared waves, which ranges from approximately 3.2 μm at 800 to 0.5 μm at 4000 cm^–1^.^32^ Consequently, the exposure of the PET layer—considering that an intact PSf layer is approximately 10 μm thick—suggests that in certain regions the PSf layer has been entirely or nearly completely removed. This finding aligns with visual observations of membrane surface cracks (Figure 2d) and SEM analysis (Figure 3h), which clearly showed the PET layer exposed after the complete removal of the PA and PSf layers. This evidence demonstrates that ozone is capable of removing both the PA and PSf layers, paving the way for innovative membrane recycling processes that are fundamentally different from those known to date.

**Figure 5:**
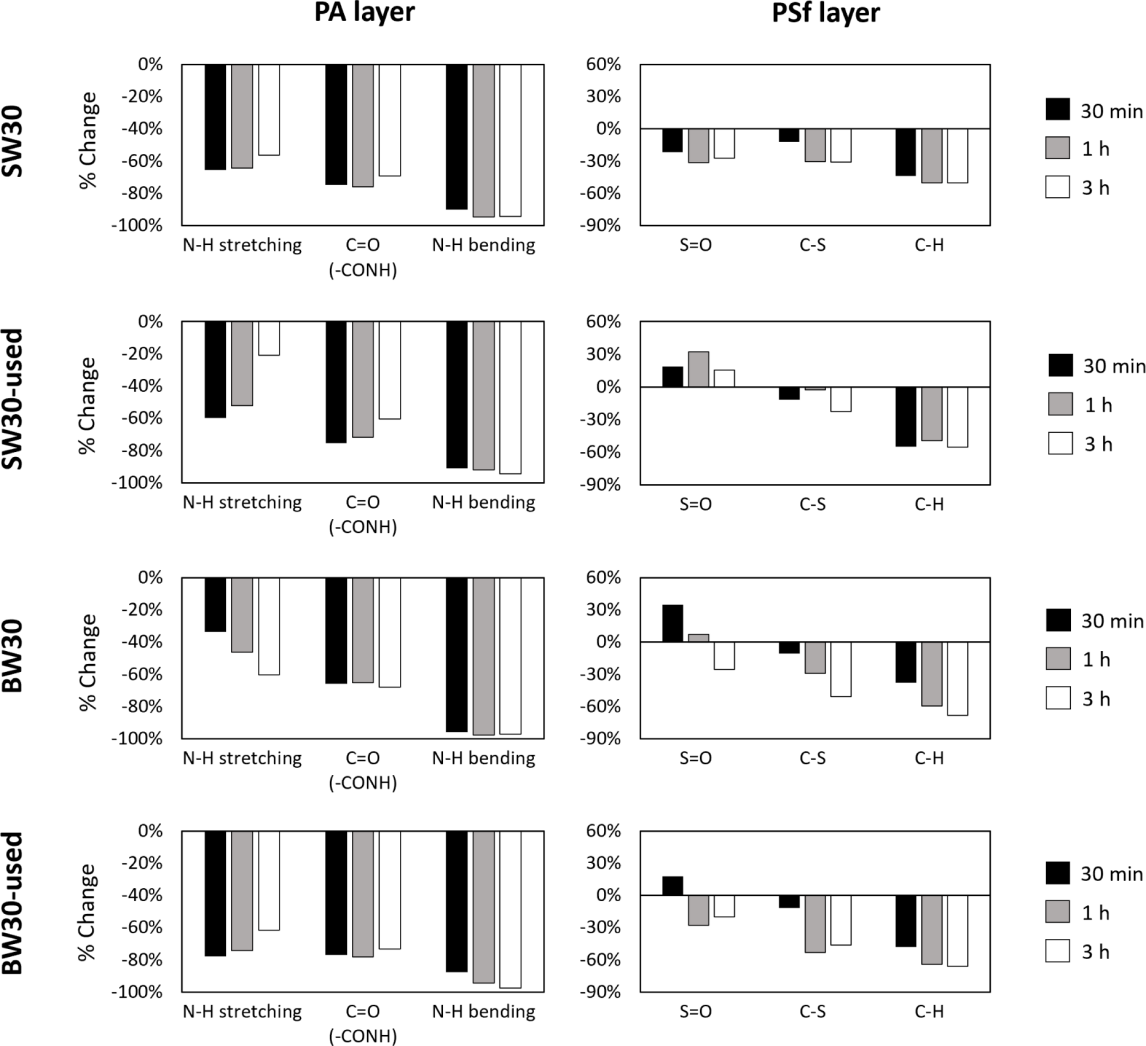
FTIR changes (compared to the initial membranes) in the studied bands for each membrane and layer during different ozone exposure times. Raw FTIR spectra are included in Figure S1.

The results also indicate that the PA layer in SW membranes is thicker than that in BW membranes. To support this observation, the ratio between the amide I and II bonds and the characteristic peaks of the PSf layer was calculated. For the new membranes, the SW membrane exhibited a ratio approximately 30% higher than that of the BW membrane. A similar trend was observed in the used membranes, with the SW membrane showing a ratio around 10% higher compared to that of the BW membrane. This fact aligns with the results obtained, suggesting that BW membranes are more sensitive to ozone treatment because of the thinner PA layer. On the other hand, when comparing new and used membranes, results show different trends probably related to the pressure applied during the membranes’ first use. The SW30-used membrane showed a thinner PA layer, likely due to the high operating pressures experienced during its operational life.

### 3.2 Ozone Low-Exposure-Time Membrane Conversion

Following the initial tests, which revealed the significant impact of ozone on the studied membranes, the ozone exposure concentration and contact duration were reduced for further analysis. New and used RO membranes, as well as new NF membranes, were subjected to ozone treatment for 1-, 5-, 15-, and 30-min, maintaining an ozone concentration of approximately 3 ppm. The membrane coupons were processed using the setup described in Section 2.2.1, and after treatment, they were characterized using the method detailed in Section 2.4.1. The resulting permeability and salt rejection data are presented in Figure 6.

**Figure 6:**
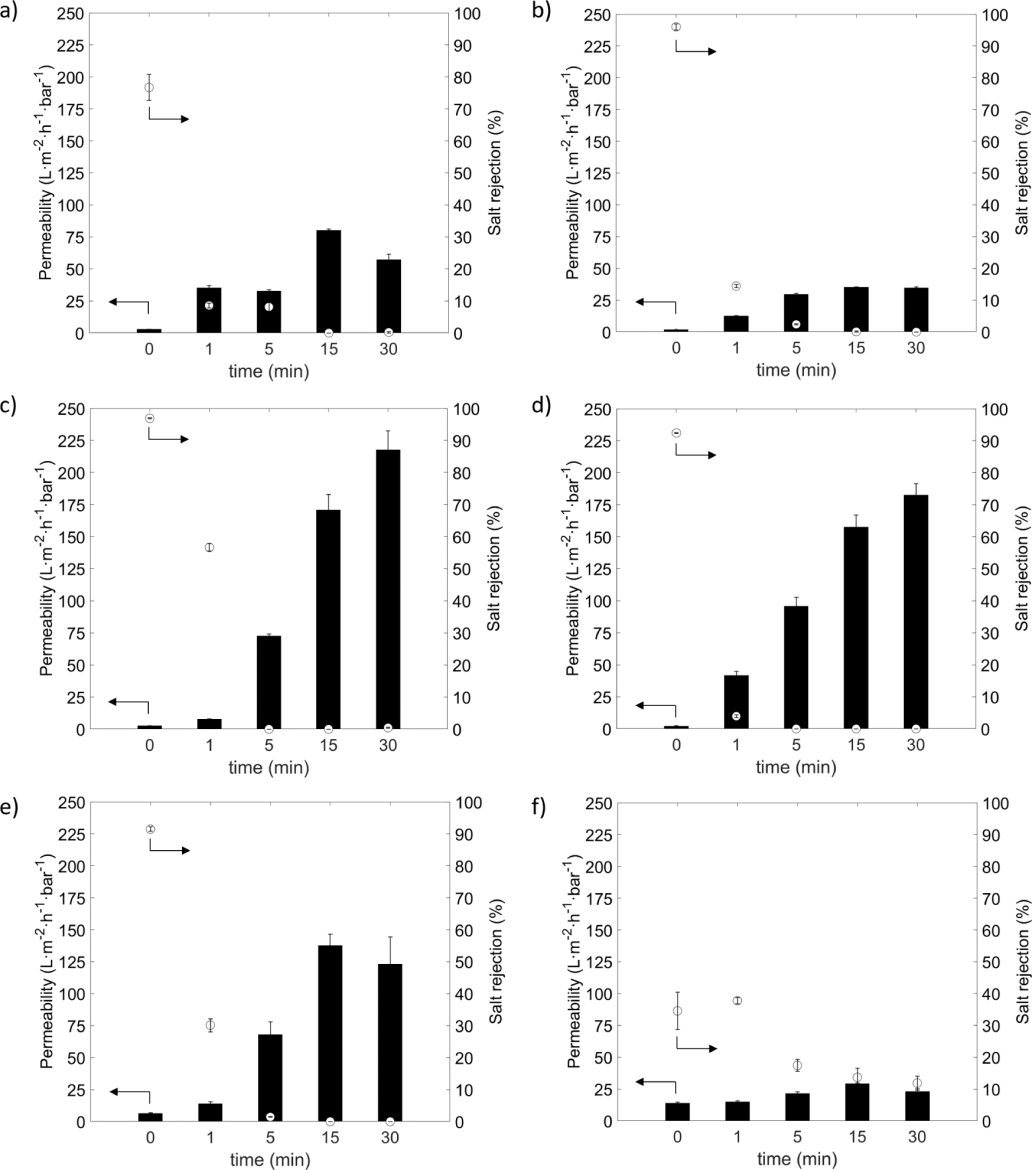
Permeability (black bar) and salt rejection (white dot) following ozone treatments: (a) SW30, (b) SW30-used, (c) BW30, (d) BW30-used, (e) NF90, and (f) NF270.

Analysis of the SW membranes (Figure 6a,b) revealed a significant increase in permeability accompanied by a sharp decrease in salt rejection as the ozone exposure time progressed. After 15 min of treatment, the new SW membrane reached a permeability of approximately 75 L·m^–2^·h^–1^·bar^–1^, while the used SW membrane exhibited a slightly lower permeability of around 50 L·m^–2^·h^–1^·bar^–1^. However, both membranes completely lost their ability to reject salt at this stage, indicating severe damage to the selective PA layer. In contrast, the BW membranes (Figure 6c,d) showed even more pronounced changes under similar conditions. After 15 min of ozone treatment, permeability values reached 175 L·m^–2^·h^–1^·bar^–1^ for the new BW membrane and 150 L·m^–2^·h^–1^·bar^–1^ for the used BW membrane. These values align with the ones obtained in previous studies^4^ for RO membranes converted into UF-like membranes. Notably, the salt rejection capacity of the BW membranes was compromised much earlier than that of the SW membranes, with a complete loss of salt rejection observed after just 5 min of exposure. This suggests that BW membranes were less resistant to ozone degradation compared to the SW membranes. As previously hypothesized, variations between RO membranes may be attributed to differences in the cross-linking degree of the PA layer, which are associated with a higher concentration of −OH groups.^30,31^ Additionally, differences between new and used membranes could result from the impact of high pressure, which may lead to membrane compaction.

Differences were also observed between the two NF membranes. The NF90 membrane (Figure 6e) exhibited behavior similar to the BW membranes, both having a fully aromatic PA layer,^19,33–35^ with a strong increase in permeability, reaching nearly 150 L·m^–2^·h^–1^·bar^–1^ after 15 min of ozone exposure. However, NF90 completely lost its salt rejection capability after just 5 min of treatment, indicating significant damage to the selective layer, as observed in BW membranes. In contrast, the NF270 membrane (Figure 6f) demonstrated greater resistance to ozone degradation compared to the other membranes studied. NF270 maintained its salt rejection capacity even after 30 min of ozone exposure and showed a much lower permeability increase, i.e., up to 35 L·m^–2^·h^–1^·bar^–1^ after 15 min of treatment. Higher resistance can likely be attributed to the semiaromatic nature of the NF270 membrane, whereas the NF90, BW30, and SW30 membranes are fully aromatic.^19,33–35^ Since ozone primarily interacts with electron-rich functional groups, the semiaromatic structure of NF270 may offer greater stability under ozone exposure.^19^

SEM and profilometry analyses were conducted on membranes exposed to 15 min of ozone treatment, as shown in Figure 7. The SW membranes exhibited partial peeling of the PA layer, whereas the BW membranes presented visible cracks across the membrane layers. These observations are consistent with the permeability and salt rejection results, which indicated a more severe impact on BW membranes compared to SW membranes. This behavior is likely related, as previously mentioned, to the thinner PA layer in BW membranes. NF90 also showed signs of PA layer removal, in line with the observed increase in permeability and decrease in salt rejection. Remarkably, NF270 displayed a severely damaged structure under SEM analysis, suggesting structural failure; however, such damage was not evident in the profilometry and performance measurements. This discrepancy suggests that ozone exposure may affect the membrane surface in a nonhomogenous way, with some regions being significantly degraded while others remain largely unaffected.

**Figure 7:**
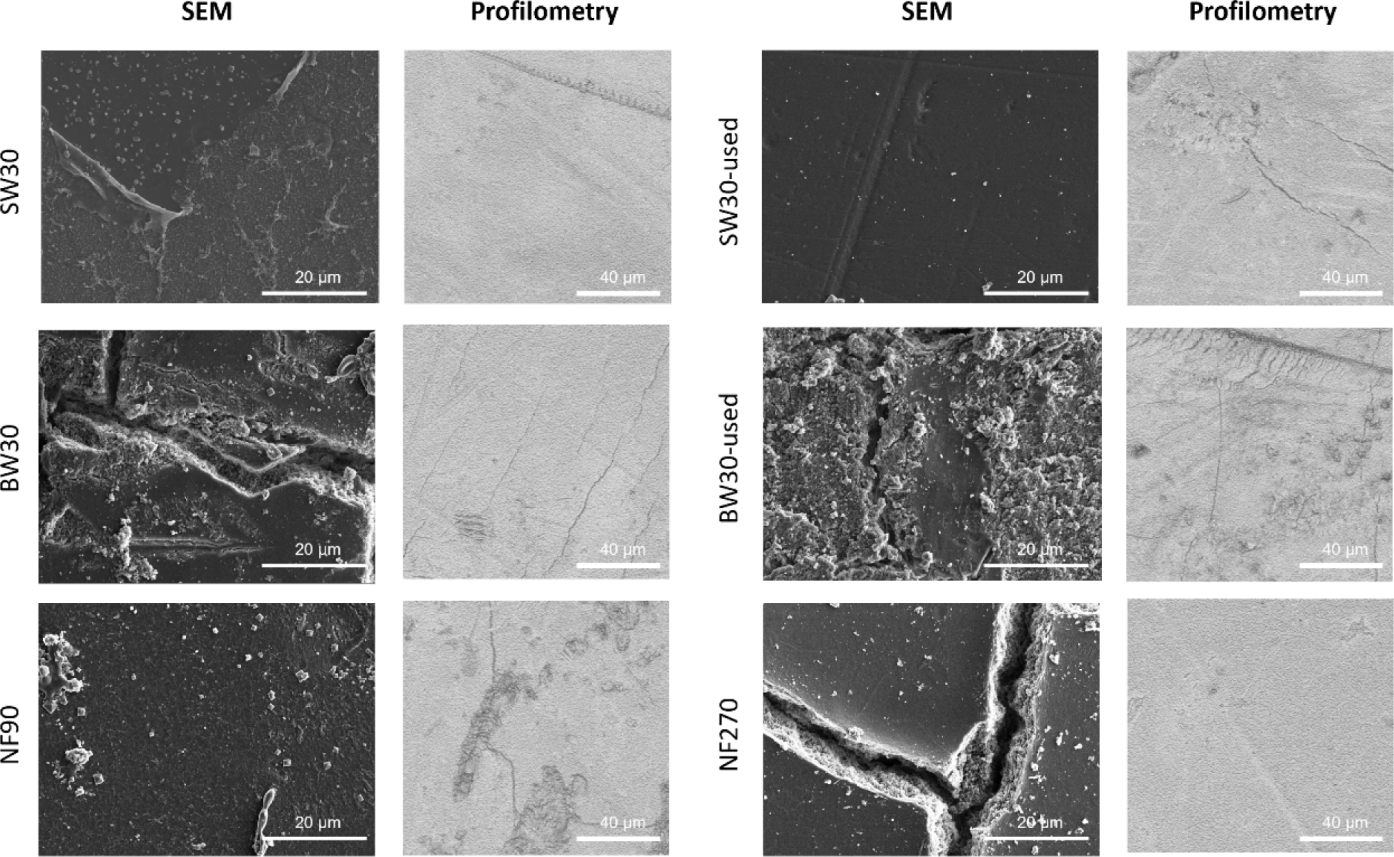
SEM and profilometry results after 15 min of low-exposure treatment for each membrane.

Profilometry calculations were conducted on the same sample at three different points, both before and after a 15 min ozone treatment, on small (5 × 5 μm) and large surface areas (50 × 50 μm). Results are shown in Figure 8. Initial results at a small scale indicated similar surface roughness across all membranes studied, with minimal change observed post-treatment. However, at a larger scale, significant differences emerged after treatment, with used membranes showing roughness values nearing 500 nm. NF90 exhibited a notably heightened roughness post-treatment, increasing from 115 to 850 nm, making it the most affected membrane. In contrast, NF270 demonstrated similar roughness levels post-treatment, highlighting ozone’s lesser impact on semiaromatic membranes.

**Figure 8:**
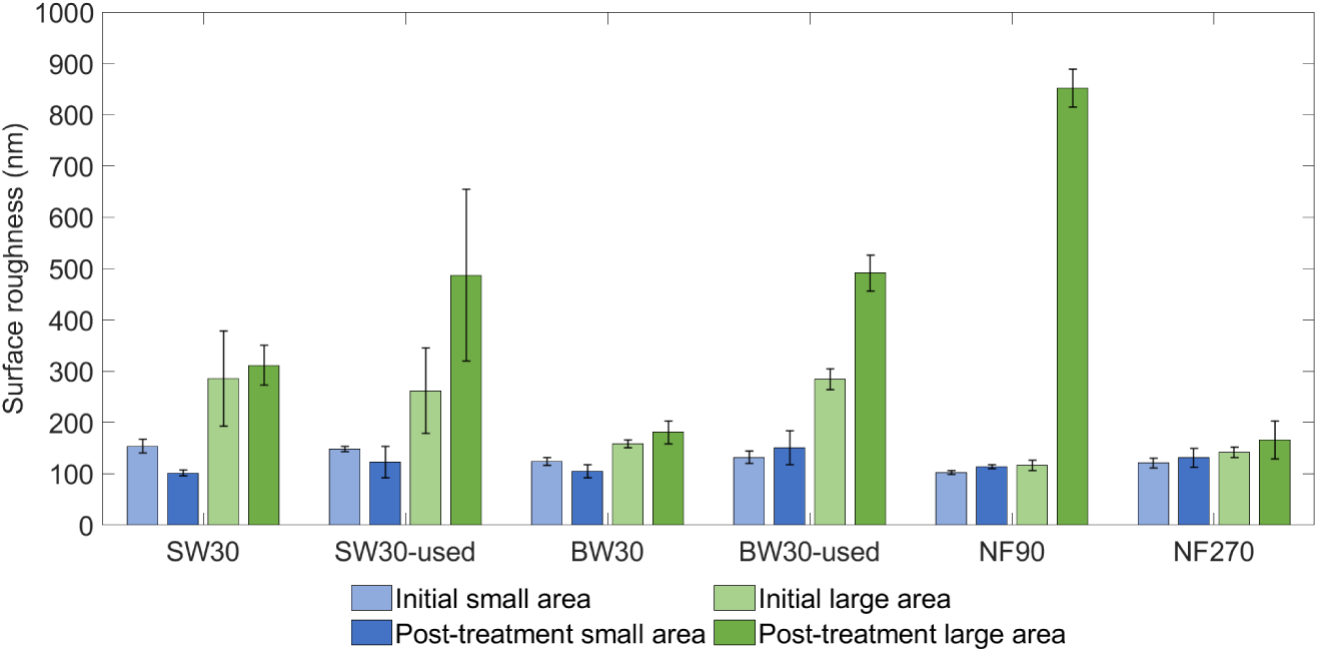
Surface roughness values for the studied membranes at small (blue) and large (green) surface areas, before (light color) and after (dark color) the 15 min ozone treatment.

### 3.3 Comparative Analysis of Ozone and Chlorine Treatment for Membrane Conversion

Ozone and chlorine treatments were then compared as methods to convert RO and NF membranes. Figure 9 shows the permeability results obtained for each membrane. Clear differences between the two approaches were observed, with ozone exhibiting a significantly higher oxidative capacity and achieving faster conversion rates at lower doses compared to chlorine.

**Figure 9:**
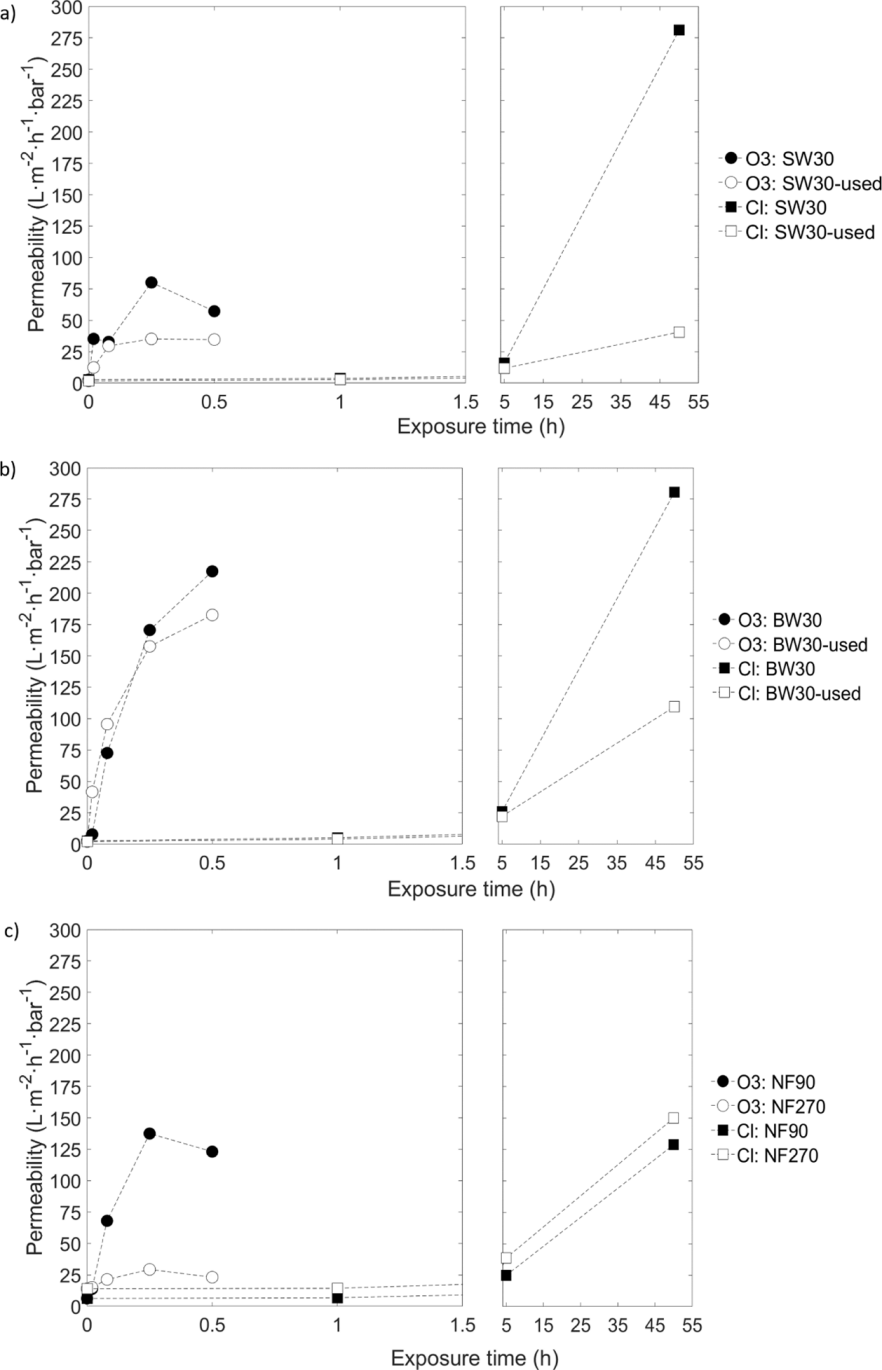
Permeability of the studied membranes following ozone (circles) and chlorine (squares) treatments: (a) SW30 (filled dots) and SW30-used (empty dots), (b) BW30 (filled dots) and BW30-used (empty dots), and (c) NF90 (filled dots) and NF270 (empty dots).

After 300,000 ppm·h of free chlorine exposure (50-h treatment at 6000 ppm), the permeability of new RO membranes increased dramatically, reaching 275 L·m^–2^·h^–1^·bar^–1^. In contrast, the ozone treatment exhibited distinct effects on the two types of RO membranes. For SW membranes, permeability reached approximately 75 L·m^–2^·h^–1^·bar^–1^, while for BW membranes, it reached 225 L·m^–2^·h^–1^·bar^–1^ after 1.5 ppm·h of exposure (30 min treatment at 3 ppm). For the used membranes, free chlorine treatment resulted in permeabilities of 40 L·m^–2^·h^–1^·bar^–1^ for SW30-used membranes and 110 L·m^–2^·h^–1^·bar^–1^ for BW30-used membranes. Under ozone treatment, the permeabilities achieved were 35 and 180 L·m^–2^·h^–1^·bar^–1^ for SW30-used and BW30-used membranes, respectively. Consistent with previous observations, BW membranes exhibited greater degradation, highlighting their lower resistance to both chemical oxidants compared to that of SW membranes. As hypothesized previously, the results suggest that the PSf layer in SW30-used membranes is more affected by industrial usage than that in BW30-used membranes. This difference may be attributed to the higher pressure applied during initial use—typically 70 bar for SW membranes versus 20 bar for BW membranes. The higher operating pressure could likely cause greater membrane compaction, which may reduce the filtration capacity and alter interactions with oxidizing agents.

The NF90 membrane exhibited a similar behavior to RO membranes under both oxidizing treatments. After exposure to 300,000 ppm·h of free chlorine, NF90 reached a permeability of nearly 130 L·m^–2^·h^–1^·bar^–1^ while with ozone treatment, a permeability of 123 L·m^–2^·h^–1^·bar^–1^ after 1.5 ppm·h was reached, demonstrating again the strong oxidizing capacity of ozone versus free chlorine. However, NF270 showed a completely different behavior, achieving a permeability of 150 L·m^–2^·h^–1^·bar^–1^ with the free chlorine treatment, and just 23 L·m^–2^·h^–1^·bar^–1^ with the ozone one. These results underscore the heightened vulnerability of the NF270 membrane to prolonged free chlorine exposure compared to that of ozone exposure. While ozone primarily targets electron-rich functional groups,^19^ free chlorine reacts more extensively with the functional groups in the PA layer such as amide I and amide II.^6^

A key distinction between the two methods lies in the layers affected during degradation. Chlorine selectively targets the PA layer, while ozone attacks both the PA and PSf layers. This observation is further supported by the liquid–liquid perm-porometry analysis shown in Figure 10. After chlorine treatment, the membrane pore size distribution remained within the range of 0–0.015 μm. In contrast, ozone treatment results in a broader pore size distribution, up to 0.07 μm for SW membranes and 0.02 μm for BW membranes, indicating the removal of the PSf layer, as well. This difference presents new opportunities for membrane recycling that have not been explored until now. Compared to the established free chlorine degradation protocol,^3,4^ which primarily degrades the PA layer, ozone treatment enables the removal of both PA and PSf layers, exposing the underlying PET support layer. The recovery of the PET layer holds significant potential for reuse in the production of new membranes, reducing the demand for virgin PET material and promoting more sustainable membrane manufacturing practices.

**Figure 10:**
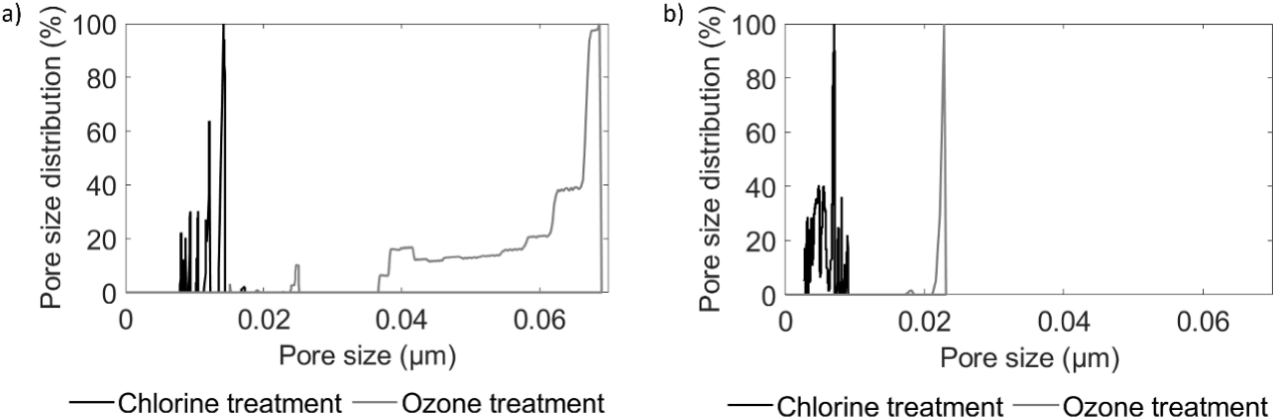
Liquid–liquid perm-porometer test for the (a) SW and (b) BW after 50 h of chlorine (black) and 15 min of ozone treatment (gray).

Differences in the degradation uniformity between the two methods are presented in Figure 11. Chlorine exposure results in a more homogeneous attack across the membrane surface, leading to consistent and predictable degradation. In contrast, ozone degradation is highly localized, occurring primarily in regions where ozone bubbles interact directly with the membrane surface. This localized effect means that ozone treatment outcomes are influenced by bubble distribution and the availability of the selective layer for ozone interaction. Ensuring uniform ozone exposure is crucial for achieving consistent membrane degradation; however, using a predissolved ozone solution could help mitigate this uneven attack.

**Figure 11:**
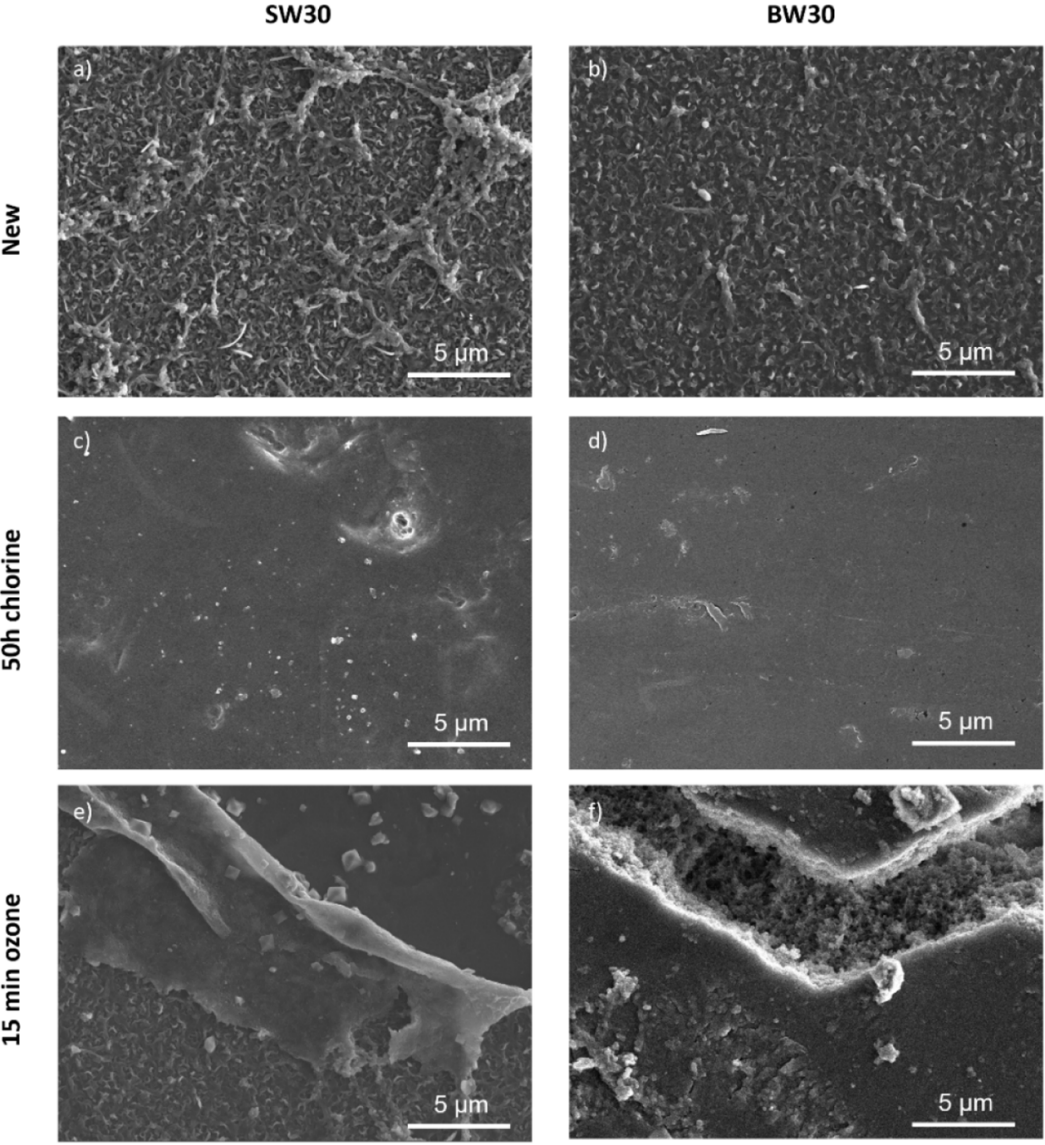
FE-SEM images of: (a,b) new SW30 and BW30 membranes, (c,d) SW30 and BW30 membranes after 50 h of treatment to 6000 ppm chlorine, and (e,f) SW30 and BW30 membranes after 15 min of treatment to 3 ppm ozone.

## 4 Conclusions

The feasibility of ozone as an alternative method to convert polymeric RO and NF membranes was systematically evaluated. A method was developed to convert new and used RO membranes into NF- and UF-like membranes, and this method was compared with the widely used free chlorine treatment. For all tested membranes, ozone exposure increased the permeability while reducing salt rejection. High ozone exposure completely removed the PA and PSf layers, resulting in visible surface cracks and offering a potential new approach to recycle the PET layer. Lower ozone exposure allowed the conversion of membranes into NF- and UF-like membranes, achieving performance comparable to that of free chlorine treatment. Ozone appeared to be more efficient, requiring less time and a lower exposure dose. The faster reaction rate of ozone offers several advantages over chlorine treatment, such as reduced processing time, lower reagent consumption, the absence of halogenated byproducts, and minimized waste. However, SEM images revealed that ozone-induced degradation was uneven and influenced by bubble distribution, whereas the free chlorine attack was uniform across the membrane surface. Thus, the implementation of membrane transformation through ozonation appears more complex. A potential solution is the use of predissolved ozone to ensure a homogeneous effect on the membrane. While further improvements to the ozone treatment method are needed, initial findings validate its potential for converting RO membranes. Future research will focus on gaining deeper insights into the polymer modifications induced by ozone oxidation, optimizing the required exposure doses, and evaluating the possibility to apply this technology at the module scale.

## Supplementary Material



## Notes

Raw FTIR spectra for each membrane new, after 15 min at low exposure and 30 min at high exposure of ozone (PDF)
