# Supplementary material for: Membrane Recycling: Exploring Ozone as a Viable Alternative to Chlorine for Polymeric Membrane Transformation
Source: ACS ES T Eng. 2025 Jul 31;5(11):3183–94. doi: 10.1021/acsestengg.5c00517 (PMC12626238; doi:10.1021/acsestengg.5c00517)
Supplement: Supplementary file 1 [file ee5c00517_si_001.pdf]

Supplementary information file for:

Title

Membrane recycling: Exploring ozone as a viable alternative to chlorine for polymeric membrane transformation

Authors

Bianca Zappulla-Sabio[a], Lide Jaurrieta[a,b], Wolfgang Gernjak[b,c], Harikrishnan Balakrishnan[d,e], Ludovic F. Dumée[f,g], Hèctor Monclús[a], Gaetan Blandin[a]

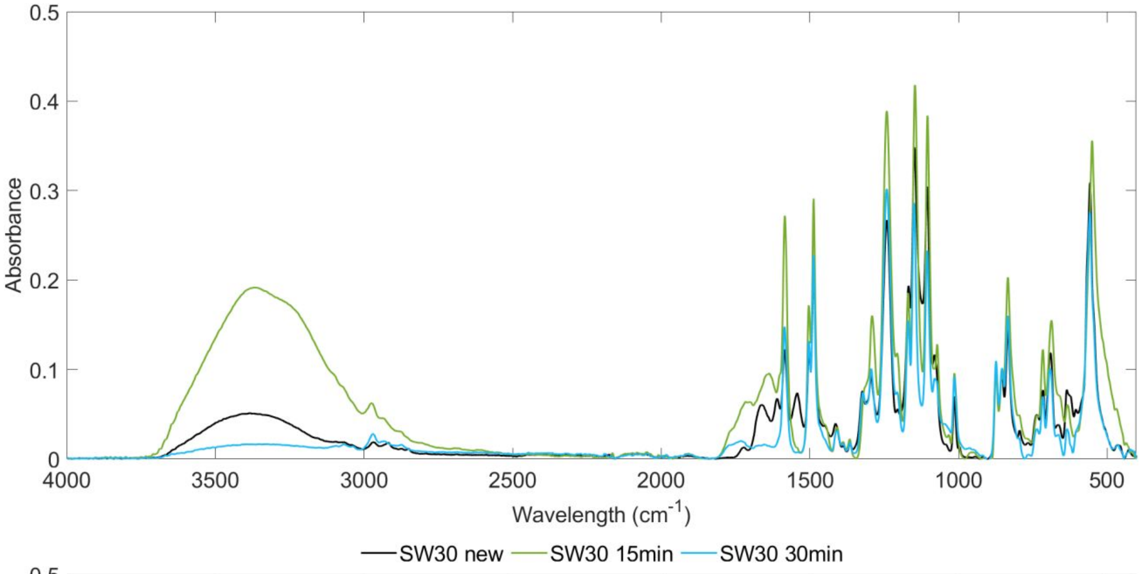
Absorbance
Wavelength (cm-1)
SW30 new
SW30 15min
SW30 30min

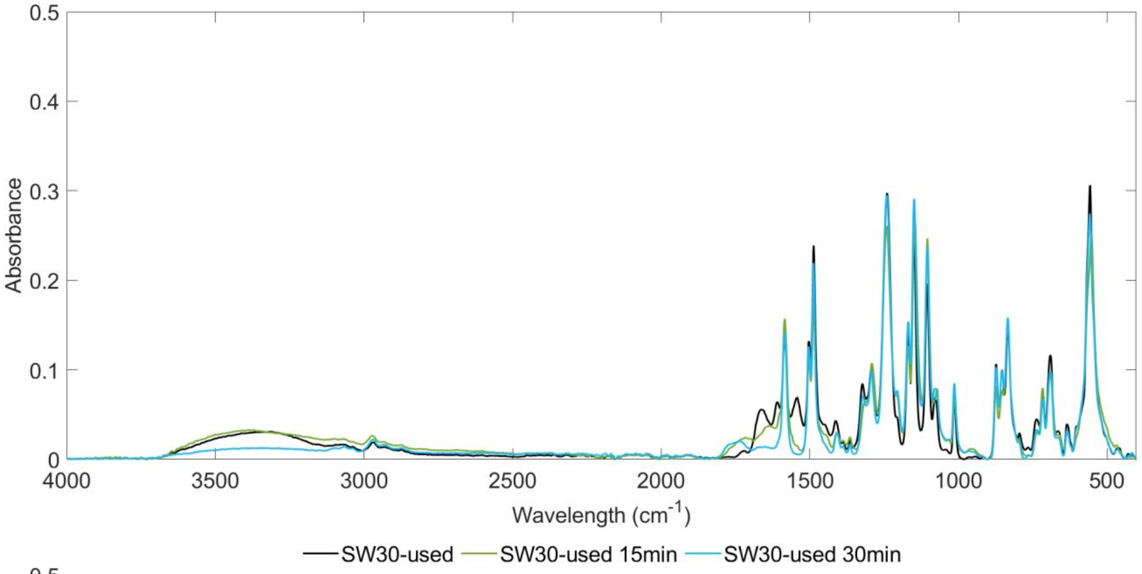
Absorbance
Wavelength (cm-1)
SW30-used
SW30-used 15min
SW30-used 30min

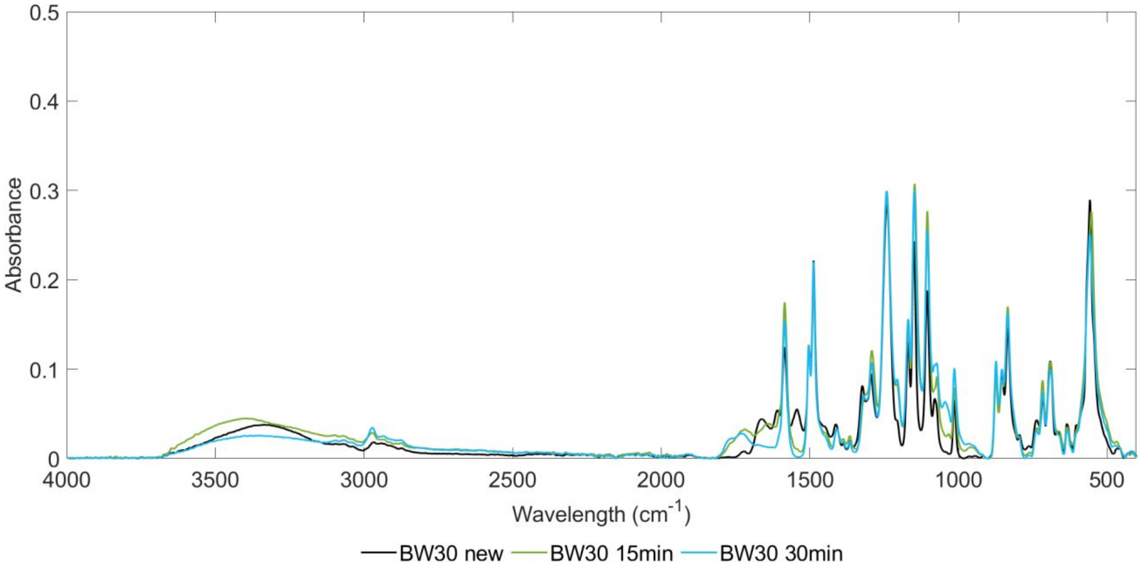
Absorbance
Wavelength (cm-1)
BW30 new
BW30 15min
BW30 30min

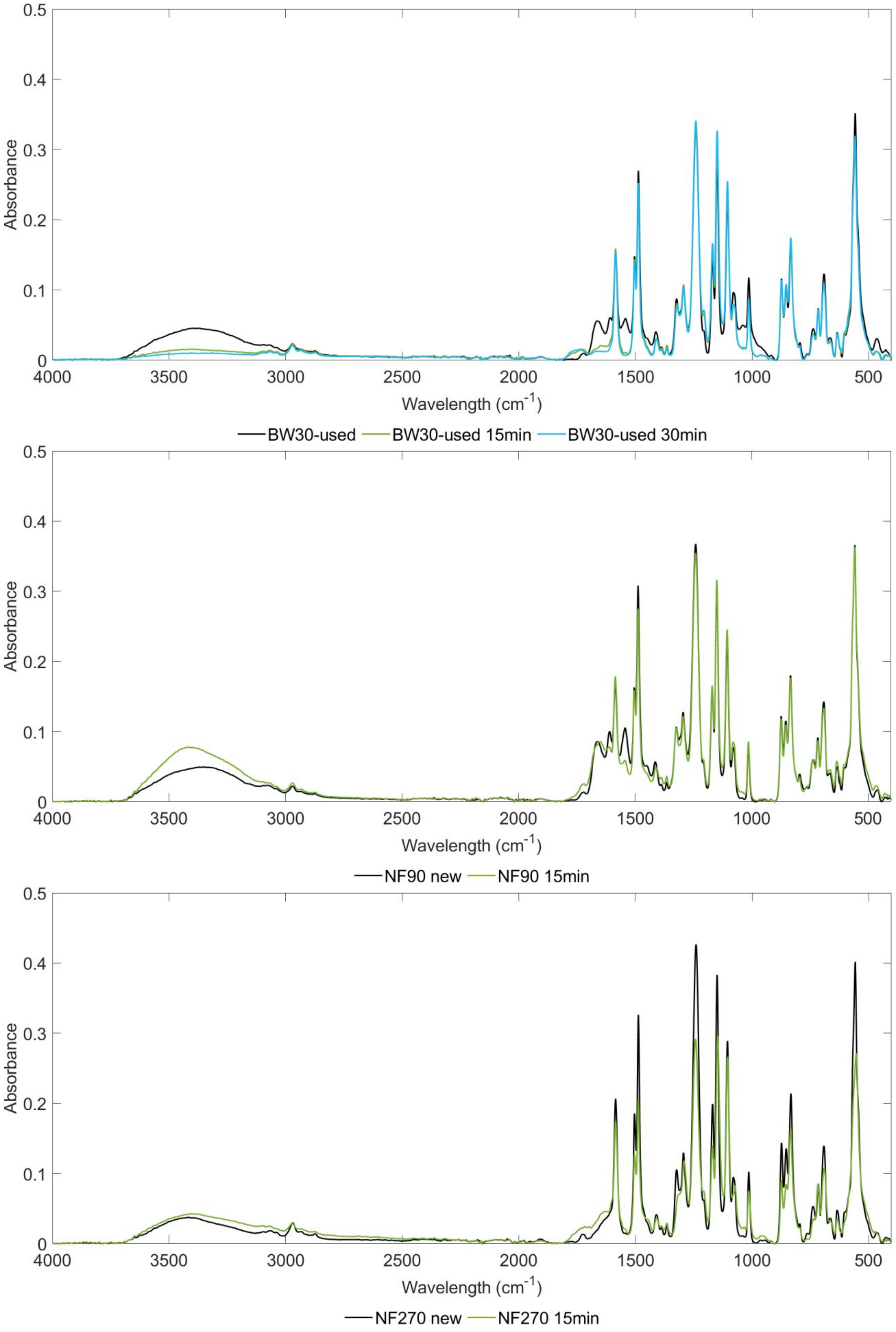

Figure S 1. Raw FTIR spectra for each membrane new, after 15-min at low exposure and 30-min at high exposure of ozone.
